# The role of non-coding RNA in ferroptosis of liver cancer and its impact on lipid peroxidation

**DOI:** 10.3389/fimmu.2025.1555518

**Published:** 2025-03-26

**Authors:** Minglu Ding, Keyuan Huo, Xiaojie Chen, Wanyao Wang, Zihan Xiang, Yidan Song, Peijian Chen, Lantao Liu

**Affiliations:** ^1^ Graduate Student Department, Mudanjiang Medical University, Mudanjiang, Heilongjiang, China; ^2^ School of Basic Medicine, Mudanjiang Medical University, Mudanjiang, Heilongjiang, China; ^3^ College of Life Science, Mudanjiang Medical University, Mudanjiang, Heilongjiang, China

**Keywords:** long non-coding RNAs, ferroptosis, liver cancer, lipid peroxidation, glutathione

## Abstract

Ferroptosis is an iron-dependent programmed death caused by the imbalance of lipid peroxides in cells. Unlike apoptosis, autophagy and necrosis, ferroptosis is mainly induced by the small molecule compound erastin. The main characteristics of ferroptosis were glutathione (GSH) depletion, inactivation of glutathione peroxidase 4 (GPX4) and reactive oxygen species (ROS) promoting lipid peroxidation. Eventually, the imbalance of lipid peroxidation regulation in cells leads to ferroptosis. The lipid metabolic pathway ultimately contributes to ferroptosis through the production of lipid peroxides. In addition, other cellular metabolic pathways can also regulate ferroptosis, such as the antioxidant metabolic pathway, which inhibits ferroptosis by clearing lipid peroxides and reducing cell membrane damage. Long non-coding RNAs (lncRNAs) are non-coding transcripts more than 200 nucleotides in length and are a less classified group of RNA transcripts that are associated with tumorigenesis and metastasis and are more tissue or cell type specific than protein-coding genes. Studies on the molecular profile of lncRNAs in plasma samples from liver cancer patients show that differentially expressed lncRNAs are mainly concentrated in biological functions related to tumorigenesis, such as cell metastasis, immune response and metabolic regulation. With different biological functions in physiological and pathological environments, the specific expression patterns of lncRNAs coordinate cell state, development, differentiation, and disease.

## 1 Introduction

Liver cancer is the sixth most common malignant tumor in the world. For patients with early liver cancer, radical treatment such as surgical resection, local ablation and liver transplantation can be performed, and the survival time of patients after surgery is almost more than 5 years. However, in reality, patients with liver cancer have reached the middle and late stages when they are first diagnosed and cannot be operated anymore (1). It is estimated that the incidence of liver cancer in East and Southeast Asia is likely to be caused by hepatitis B and C virus infections, and the incidence of liver cancer may also be related to alcohol abuse, obesity, diabetes, and aflatoxin intake (2). Despite advances in diagnosis and treatment techniques, there are currently very limited methods available clinically to prevent liver cancer recurrence and metastasis. Therefore, it is very important to study the pathogenesis of liver cancer and new therapeutic targets. It is reported that changes in iron metabolism play an important role in the pathogenesis of liver cancer, an iron-rich diet will increase the risk of liver cancer, and activating ferroptosis may prevent the proliferation of liver cancer cells, so it is possible to intervene in liver-related diseases by regulating the ferroptosis pathway (3). If iron homeostasis is disrupted, it triggers ferroptosis in the liver, where iron participates in non-enzymatic lipid peroxidation through the Fenton reaction, where Fe^2+^ is oxidized to Fe^3+^, which in turn kills cancer cells by inducing ferroptosis (4).

## 2 Ferroptosis

### 2.1 Definition and characteristics of Ferroptosis

Cell death can be performed through different subroutines. Interest in iron death has increased since it was described in 2012 as an iron-dependent form of non-apoptotic cell death (5). Ferroptosis is an iron-dependent regulatory cell death mode. The accumulation of iron ions in cells is a necessary condition for iron death. The core feature of ferroptosis is the damage of cell membrane system caused by abnormal accumulation of lipid peroxides (6, 7). Distinct from other programmed cell death forms such as apoptosis, necrosis, and autophagy, ferroptosis exhibits unique characteristics in morphology, biochemistry, and genetics. Morphologically, it is characterized by reduced mitochondrial volume, increased membrane density, and the disappearance of cristae, while nuclear morphology remains largely unchanged. Biochemically, ferroptosis is distinguished by its dependence on Fe^2+^ to catalyze lipid peroxidation via the Fenton reaction, which is accompanied by decreased GPX4 activity and imbalances in antioxidant systems, including the glutathione system (8, 9).

### 2.2 Inducible factors related to ferroptosis

#### 2.2.1 Ferroptosis induced by activating transcription factor 3 (ATF3)

The characteristics of ferroptosis are morphologically manifested as the reduction of mitochondria, the concentration of mitochondrial membrane, and the reduction or disappearance of mitochondrial ridge (10). The disorder of ferroptosis is related to various physiological and pathological processes, such as neurodegenerative diseases, acute renal failure, liver and heart injury. Although the molecular mechanism of ferroptosis is still largely unknown, some transcription factors such as ATF 3, ATF 4, YAP1, and HIF-1α have been found to play an important role in ferroptosis, and these transcription factors can regulate the expression of genes associated with ferroptosis through transcription-dependent or transcription-independent mechanisms. They may be involved in the regulation of iron ion metabolism, oxidative stress response and mitochondrial function, which may affect the ferroptosis process of cells (11). In addition, ATF 3 can also be used as a transcription factor to coordinate a variety of signal transduction pathways, such as apoptosis and cell differentiation, and is also an important link between inflammation, oxidative stress and immune response, and its expression is up-regulated under various stress conditions to maintain cell homeostasis (12). To date, a few genes have been shown to be direct transcription targets for ATF3, but ATF3 contains domains that bind to regulatory elements to inhibit or activate transcription depending on the cellular environment. In addition, ATF3 competes with transcriptional activators for BS-1 or BS-2 binding sites to inhibit the SLC7A11 promoter, which, since the SLC7A11 promoter is positively correlated with the XC^-^ system, inhibits system XC^-^, depletes intracellular GSH, and ultimately promotes Erastin-induced ferroptosis (13). Sorafenib is a molecular targeted drug for the treatment of advanced hepatocellular carcinoma and an effective inducer of ferroptosis, but its clinical application is limited due to cardiotoxicity. A comparison with GEO database data showed that the expression of ATF3 was significantly increased in sorafenib treated human cardiomyocytes, and high expression of SLC7A11 protected cells from ferroptosis. Knocking down SLC7A11 sensitised cardiomyocytes to ferroptosis caused by sorafenib. In conclusion, ATF3 can promote ferroptosis by inhibiting SLC7A11, and can also affect the efficacy of sorafenib by regulating the expression of SLC7A11, which has an important impact on the treatment of tumors (14).

#### 2.2.2 p53-mediated ferroptosis

**Figure 1:**
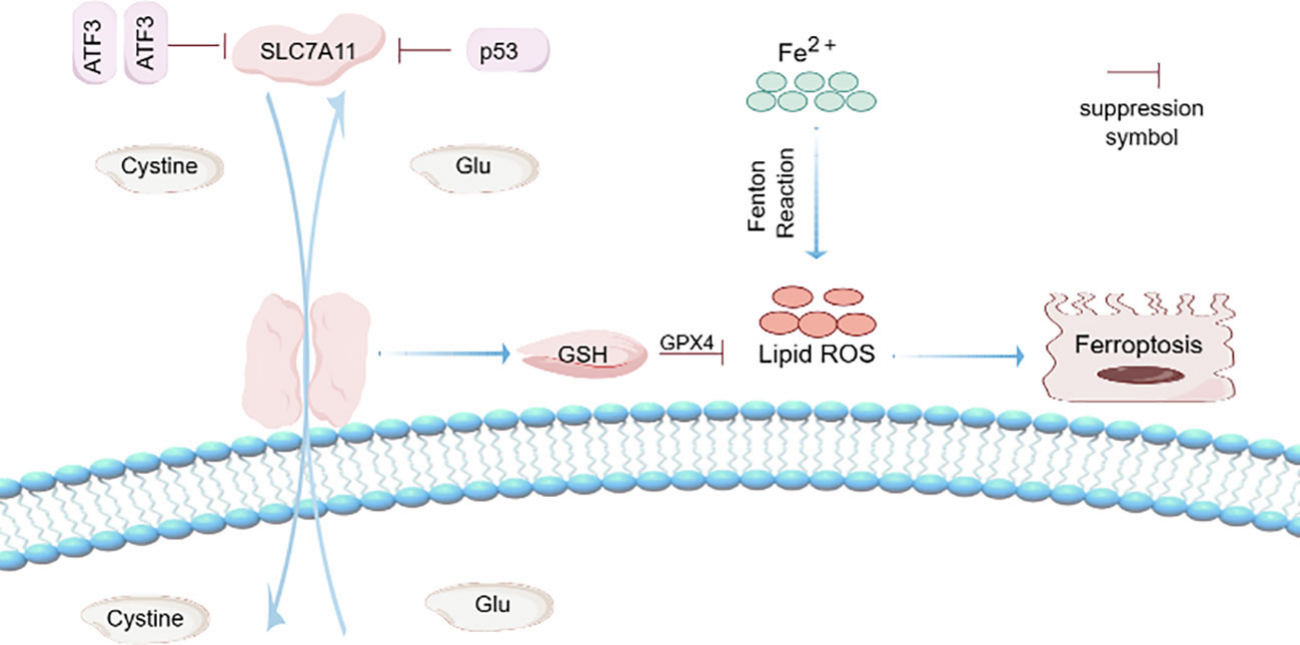
Ferroptosis mechanism. Activating transcription factor 3 (ATF3) competes with transcription activator BS-1 or BS-2 binding sites to inhibit SLC7A11 promoter. Since SLC7A11 promoter is positively correlated with XC- system, inhibits system XC-, consumes intracellular GSH, and ultimately leads to ferroptosis. ATF3 promotes iron-free cell death by inhibiting the expression of SLC7A11. Known as the “guardian of the genome,” p53 can suppress tumors by inducing aging and programmed cell death. p53 activation is not a simple all or nothing pattern, but a dynamic process. p53 can inhibit the expression of SLC7A11 through transcriptional mechanism, reduce cystine intake, and induce ferroptosis. In addition, p53, as a regulator of ferroptosis, can also directly regulate the metabolic diversity of cells by promoting mitochondrial respiration and induce the production of ROS. Fe^2+^ can induce Fenton Reaction and induce the production of ROS, thus promoting ferroptosis.

Known as the “guardian of the genome,” p53 gene mutations are often observed in human cancers, and p53 can suppress tumors by inducing aging and programmed cell death. In addition, p53 also has many other functions, such as promoting DNA repair, regulating cell metabolism and participating in inflammatory responses (15). The p53 protein, discovered in 1979 and encoded by the tumor protein p53 (TP53 or p53) gene, attracted the attention of the cancer research community and the pharmaceutical industry, making it the most widely studied gene. The activation of p53 is not a simple all-or-nothing pattern, but a dynamic process. Cell heterogeneity, stress characteristics, multiple regulatory factors and the stability of target genes jointly determine the dynamic change of p53 activity (16). In addition, p53 protein is also an important tumor suppressor protein in human cells. In order to ensure the function of p53 protein in the regulation of cells, its level and activity are strictly regulated in cells. When p53 protein is usually maintained at a low level in normal cells, the half-life of p53 protein will increase significantly. A variety of intracellular and extracellular stress signals (such as DNA damage, hypoxia, nutrient depletion, oncogene activation, etc.) accumulate in the cell. Once these stress signals are activated, p53 binds to the response elements in its target genes to regulate their expression in a transcriptional manner (17). The p53 tumor inhibition pathway is a very key signal transduction pathway in cell biology, mainly regulating cell cycle, DNA repair, and gene expression through p53 protein, so as to effectively prevent the occurrence and development of cancer. A recent study showed that p53 can inhibit SLC7A11 expression through transcriptional mechanisms, reduce cystine intake and induce ferroptosis in cancer cells (18). In addition, p53, as a regulator of ferroptosis, can also directly regulate the metabolic diversity of cells by promoting mitochondrial respiration and induce the production of ROS (19), and excessive production of ROS will trigger p53-mediated ferroptosis (20) (**Figure 1**).

#### 2.2.3 Autophagy and ferroptosis

Cell death was divided into apoptosis, necrosis, and autophagy based on morphological criteria (21). Autophagy is a natural, regulated and destructive biological process, which has the function of decomposing unnecessary or dysfunctional components in cells. Autophagy pathway is an important degradation and recycling system in cells, which plays an important role in maintaining intracellular stability and regulating cell growth (22). Impaired autophagosome maturation has been implicated in the pathogenesis of various human diseases, such as metabolic diseases, cancer and myopathy (23). Both genomic and epigenetic factors can regulate autophagy in liver cancer, and autophagy is also evident in its ability to promote ferroptosis in liver cancer cells (24). The successive stages of autophagy include inducing the formation and eventual degradation of cells, phagocytes, autophagosomes and autolysosomes. In addition, autophagy can also inhibit or promote cell death, thereby regulating the fate of liver cancer cells (24). To investigate whether Erastin-induced lipid peroxidation is dependent on the autophagy pathway, wild-type fibroblasts were treated with autophagy inhibitors in the absence or presence of erastin. The results showed that autophagy can actively regulate Erastin-triggered cell ferroptosis and increase lipid peroxidation. Depletion of autophagy attenuated lipid peroxidation in erastin induced ferroptosis and decreased cell sensitivity to ferroptosis (25). Some studies have also shown that in liver cancer cell lines, the RNA-binding protein chaperone of NOB 1 (PNO 1) plays an important role in the reprogramming of GSH metabolism by promoting autophagy, and inhibition of PNO 1 can inhibit the transcription of SLC7A11 through p53, thereby increasing the sensitivity of tumor cells to ferroptosis. Promotes ferroptosis in tumor cells (26).

### 2.3 Role of ferroptosis in tumor

**Figure 2:**
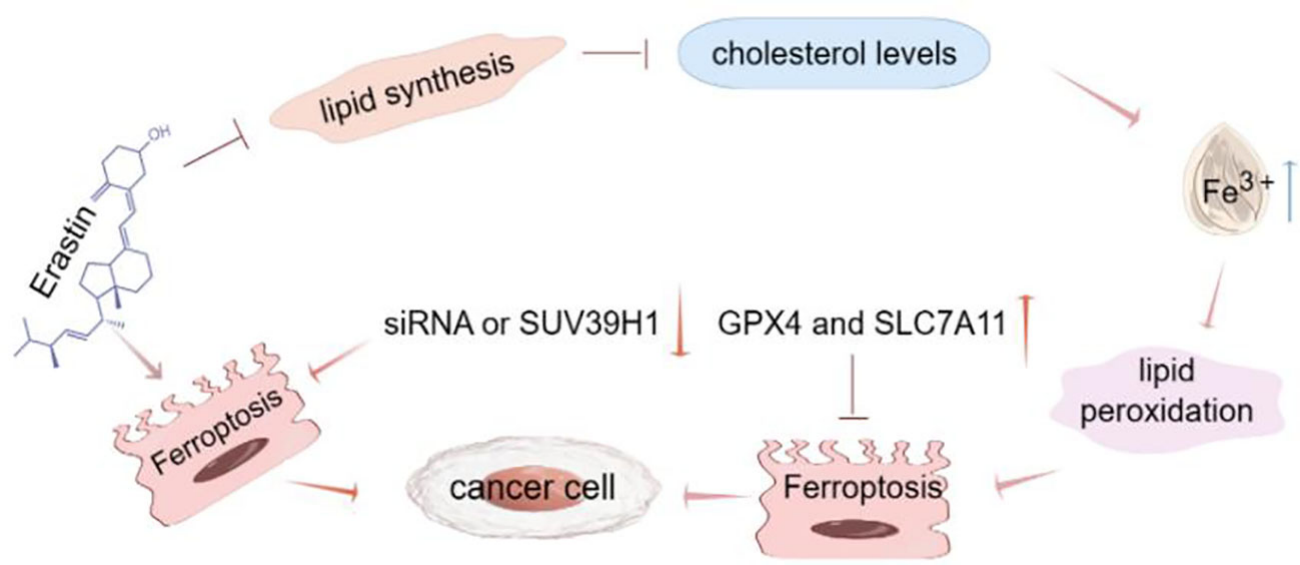
Ferroptosis and tumor. Ferroptosis has emerged as a promising anti-tumor treatment, and targeting ferroptosis to kill tumors is seen as a potentially effective strategy. Erastion can induce ferroptosis and inhibit the lipid synthesis, thereby inhibiting cholesterol. Low cholesterol level promotes the increase of Fe^3+^ content, which leads to the development of lipid peroxidation, thereby leading to ferroptosis. However, increased expression or activity of GPX4 and SLC7A11 in the ferroptosis pathway can also inhibit ferroptosis. There are also multiple tumors associated with ferroptosis that induce iron accumulation and lipid peroxidation by knocking down siRNA or SUV39H1 leading to ferroptosis.

Ferroptosis has emerged as a promising approach for anti-tumor therapy, and targeting ferroptosis to kill tumors is seen as a potentially effective strategy, such as Lipocalin-2 (LCN2), a protein found in the human body that plays a role in multiple biological processes, including inflammation, immune response, and lipid transport. Anti-lcn2 therapy is a treatment that targets the LCN2 protein and improves liver cancer treatment by targeting ferroptosis (27). Inducing ferroptosis could also synergistically enhance the effects of immunotherapy, paving the way for future combination treatment approaches (28). As a congenital tumor suppressive mechanism, ferroptosis is involved in the biological process of tumors, mainly existing in the small mitochondria (29). For example, GPX4 enters the mitochondria via the mitochondrial protein input system, the outer membrane translocase/inner membrane translocase (TOM/TIM) complex, and then degrades GPX4 mainly through mitochondrial autophagy and ROS induced damaged mitochondria, leading to ferroptosis in hepatocytes (30). The co-regulation of iron accumulation, lipid peroxidation and antioxidant mechanism enables tumor cells to avoid ferroptosis, thus exhibiting infinite proliferation of tumor cells. Increased expression or activity of GPX4 and SLC7A11 in the ferroptosis pathway can promote tumor proliferation by down-regulating ferroptosis (29). There are also a variety of tumors associated with ferroptosis, such as renal cell carcinoma, cervical cancer and other prone to ferroptosis, the anti-tumor effect of ferroptosis has been widely studied in variant cancers, and is regarded as the Achilles heel of almost untreatable tumors (31). Renal clear cell carcinoma is a common renal malignant tumor with a poor prognosis. It induces iron accumulation and lipid peroxidation by knocking down siRNA or inhibiting the heterogeneous inhibitory factor 3e9 homolog 1 (SUVs 39 H1), leading to ferroptosis and disrupting the growth of renal clear cell cancer cells (32). Cervical cancer is one of the most common malignant tumors in women, and chemotherapy is the main treatment for cervical cancer, which plays an important role in improving patient survival by inducing cancer cell death (33). Although ferroptosis effectively enhances cancer immunotherapy, inducing ferroptosis may impair T cell survival, research has found that there is a new cancer therapy called FAST, Combining iron oxide nanoparticles with cancer-selective knockout of seven key iron-death resistance genes (FPN, LCN2, FTH1, FSP1, GPX4, SLC7A11, and NRF2), FAST was found to have significant anti-tumor activity in a variety of cancer cells, with little effect on normal cells. Succeeded in turning a common iron nanomaterial into an unprecedented cancer killer (34) (**Figure 2**). Overall, ferroptosis makes us fully expect it to provide a new anti-tumor treatment. Recent studies have shown that cancer cells with a high mesenchymal state have become an important mechanism for the acquisition of targeted therapies and new drug resistance. This drug-resistant mesenchymal cancer cell produces a state of non-oncogene addition in GPX4, and this inhibition intuitively leads to ferroptosis. Consistently, persistent cancer cells nominated to escape from conventional cytotoxic therapy via dormant tumors showed the same selective dependence on the GPX4 pathway. Therefore, ferroptosis may be considered a viable treatment strategy for reversing cancer treatment resistance strategies (35).

### 2.4 Ferroptosis and liver cancer

The liver is located at the junction of the portal vein and systemic blood flow, and is an organ with high iron content in the human body (36), which is crucial for maintaining systemic iron homeostasis, which can cause pathological changes in the liver, such as liver fibrosis, if destroyed. In the pathogenesis of liver fibrosis, transforming growth factor β1 (TGF-β1) is highly expressed (37), and truncated transforming growth factor β-receptor II (TβRII) can inhibit the highly expressed TGF-β1, thus blocking the activity of TGF-β1 in liver fibrosis, which is a drug for the treatment of liver fibrosis (38). The main regulatory mechanism of ferroptosis in hepatocytes is to trigger cellular oxidative stress and lipid peroxidation, the regulatory mechanism of ferroptosis in hepatocytes, a cellular process that triggers oxidative stress and lipid peroxidation, DNA damage, and cell death, specifically induces CXCL 10-β-dependent activation of the recruitment of CD8 + T cells, and finally re-enhances the anti-tumor capacity of the adaptive immune response (39). Donafinil is a multi-receptor tyrosine kinase inhibitor used in the treatment of liver cancer patients (40). The ATF4 is a family member of ATF, and ATF4 can also slow down the occurrence of liver cancer (41). The mRNA expression levels of five genes in the ferroptosis pathway (GPX4, SLC7A11, AIFM2, ACSL4, Nrf2) in liver cancer tissues were higher than those in normal tissues, especially the high expression of GPX4 was closely related to liver cancer patients, and the high expression of GPX4 increased the progression-free survival of liver cancer. It plays a negative regulatory role in the occurrence and development of liver cancer and promotes the development of cancer (42). In addition, ferroptosis is strictly regulated by two types of genes with opposite functions, namely HIC1 and HNF4A transcription factors, which are negatively correlated with the tumor stage of liver cancer. In liver cancer, the phosphorylation level of HIC1 will increase, thus promoting the development of tumor, and disrupting the balance between HIC1 and HNF4A is conducive to the treatment of liver cancer (43).

## 3 Cellular mechanisms related to liver cancer

### 3.1 Cell proliferation

In multicellular organisms, cell proliferation is realized through the regulation of cell cycle, which is a complex process involving multiple steps such as DNA replication, chromosome allocation and cell division. The imbalance of hepatocyte proliferation mechanism is one of the important factors in the occurrence of liver cancer. The overexpression of HULC RNA in liver cancer tissues promotes the proliferation, migration and invasion of liver cancer cells *in vitro*, and promotes the growth of xenograft tumors *in vivo*. miR-2052 is a microRNA. Compared with non-cancer tissues, the level of miR-2052 in liver cancer tissues is reduced, which inhibits the proliferation of liver cancer cells. HULC can also act as a sponge of miR-2052 in liver cancer cells. The epithelial transformation factor MET is the direct target of miR-2052 and is positively correlated with HULC expression, while the expression of miR-2052 is negatively correlated with HULC expression. Mechanistically, high levels of HULC promote MET expression via sponge miR-2052, which in turn promotes liver cancer growth via the miR-2052/MET axis (44). In addition, whole genome sequencing (WGS) showed that circ-ZEB 1 and PIK 3CA were also highly expressed in HCC tissues, which was associated with poor prognosis of HCC. Down-regulated CIRC-ZEB1 expression inhibited the proliferation of HCC cells and promoted apoptosis. The low expression of MIR-199a-3p in liver cancer tissues can block the effect of CIRC-ZEB1 on liver cancer cells, that is, CIRC-ZEB1 promotes the expression of PIK3CA by silencing miR-199a-3p, thus affecting the progression of hepatocellular carcinoma, and can be used as a biomarker for hepatocellular carcinoma (45).

### 3.2 Apoptosis

Apoptosis is a process of programmed cell death determined by genes, which is a key mechanism for the normal development of an organism and the stability of its internal environment. The main functions of apoptosis include the removal of redundant or damaged cells, the maintenance of tissue structure and function, the prevention and treatment of cancer and the regulation of the immune system. In liver cancer tissues, levels of lncRNA PLAC2 are lower than in non-cancer tissues, and low levels of PLAC2 are strongly associated with poor survival. p53 protein is an important factor in the inhibition of cancer, and its signal transduction can be realized through the interaction with lncRNAs, which is down-regulated in liver cancer and positively correlated with PLAC2. In addition, PLAC2 is also the upstream activator of p53, which has an inhibitory effect on tumors, and its expression in liver cancer tissues is not affected by hepatitis B and C virus infection. If PLAC2 is overexpressed, the expression of p53 will be up-regulated, thus increasing the apoptosis rate of cancer cells, while the overexpression of p53 cannot affect PLAC2. In conclusion, PLAC2 can mediate apoptosis of cancer cells through up-regulation of p53, and predict the expression of PLAC2 before treatment is conducive to the prognosis of liver cancer (46).

### 3.3 Autophagy

There is already growing evidence that anticancer drugs inhibit tumor progression by stimulating autophagy (47). During autophagy, cells wrap damaged or degraded cells in bilayer membrane structure autophagy vacuoles, which are then transported to lysosomes for degradation, releasing amino acids and metabolites that can be reused. LncRNA DCST1-AS1 was found to be an abnormally expressed gene in liver cancer tissue through gene chip screening, and the higher its expression, the worse the prognosis of patients. LncRNA DCST1-AS1, as a carcinogen of liver cancer, plays a crucial role in the regulation of liver cancer metastasis. Autophagy of hepatocellular carcinoma cells is promoted through AKT/mTOR signaling pathway, and the progression of hepatocellular carcinoma is inhibited through autophagy process. In addition, the deletion of lncRNA DCST1-AS1 in hepatocellular carcinoma cell line (HepG2) showed anti-tumor properties, accelerating apoptosis, inhibiting cell migration and stimulating autophagy in hepatocellular carcinoma cells. Therefore, lncRNA DCST1-AS1 is a potentially effective drug target for the treatment of patients with clinical liver cancer (48).

## 4 Occurrence and development of liver cancer, LncRNA and liver cancer

### 4.1 Progress of exosomes in liver cancer

Exosomes are small (~100 nm) membrane-bound extracellular vesicles released into biological fluids by various types of cells (49). Their main functions include intercellular communication, substance transport, immune regulation, tissue repair and regeneration, and for the diagnosis and treatment of diseases (50). They can be secreted by a variety of cells such as macrophages. It then migrates from macrophages to tumor cells to promote tumor progression (51), invasion, and metastasis. Tissue fibrosis and extracellular matrix (ECM) hardening can also stimulate the release of exosomes by cancer cells, ultimately promoting tumor growth. As a drug carrier, exosomes are a newly discovered cell communication tool. Almost all human cells can secrete exosomes, and tumor cells release more exosomes than normal cells. Exosomes have been used in the treatment of many diseases, such as Alzheimer’s disease, depression, Parkinson’s disease, diabetes, infectious diseases, etc. (52), and can also be used as a marker for early liver cancer screening. It has the characteristics of stable circulation, good biocompatibility, low immunogenicity and high transport efficiency, and is closely related to the occurrence, development and metastasis of tumors (53). By comparing the level of exosomal tsRNA between liver cancer patients and healthy people, it was found that the tsRNA in plasma exosomes of liver cancer patients increased significantly (54), among which miR-122 was the most reported, which can regulate the occurrence and development of liver cancer by affecting the tumor cell cycle, and block it with miR-221 as the therapeutic target (55), which can block the G1 phase of liver cancer cells. Thus, the proliferation of liver cancer cells is weakened, and the metastasis of liver cancer cells is eventually controlled (56).

### 4.2 The role of liver cancer stem cells in liver cancer

Cancer stem cells (CSC) are pluripotent subsets of cells in tumor tissues with the potential to spread, initiate and maintain tumor growth. They are also the initiation cells of cancer and play an important role in tumor growth, metastasis and treatment (57). Targeting CSC is considered an effective way to eradicate primary tumors and prevent distant metastasis of HCC (58). The CSC model states that tumor growth is driven by a subset of tumor stem cells in cancer, such as circRNA, which is a key subset with dry characteristics that promotes the development of HCC (59). The model explains several clinical observations in liver cancer as well as other cancers, including the almost inevitable recurrence of tumors after initial successful chemotherapy or radiotherapy. And the phenomenon of tumor dormancy and treatment resistance (60). Through transcriptome microarray analysis, a highly expressed long-chain non-coding RNA in liver CSC was identified as lncTCF7, which can induce liver CSC self-renewal and tumor proliferation by mediating Wnt signaling (61). Stem cell markers are specific molecules that can recognize and isolate cancer stem cells, and can be used alone or in combination. One of the most common markers in liver cancer stem cells is CD133, and CD133+HCC cells isolated from the liver cancer cell line Huh 7 show higher proliferation and tumorigenic potential. In addition, CD44 is also an important marker (62), and it has been reported that CD44 can more accurately define the surface phenotype of liver stem cells, and CD133 and CD44 double positive cells are more resistant to chemotherapy drugs. CD44 blocking prevents CD90+ cells from forming local and metastatic tumor nodules (63).

### 4.3 Breakthrough of immunotherapy in liver cancer

With the rapid development of systematic therapy for liver cancer, immunotherapy has been widely used in the treatment of liver cancer, obtaining the first FDA approval in the form of recombinant cytokines, namely interleukin-2 (IL-2) and interferon (IFN-α), and researchers found that bioengineered immune cells are prone to fatigue after attacking cancer cells. Some treatments involving immune checkpoint inhibitors (ICI), such as anti-PD-1/L1 and anti-CTLA4 antibodies (64), can prolong the survival of patients with various cancers and greatly improve the prognosis of patients with advanced liver cancer. There are two different ICI protocols, atezolizumab + beizumab and tremelimumab + durvalumab, which are approved standard first-line therapies. Their mechanism of action is to block the signal transmission pathway between cancer cells and immune cells, thereby activating the immune system. Allowing it to effectively recognize and attack cancer cells. At the same time, cancer time therapy also plays an important role in the carcinogenic process, and some factors such as administration frequency and dose, individual differences, treatment goals, etc. will affect the survival rate of advanced cancer, which should be adjusted according to the specific situation of patients (65). In short, the breakthrough of immunotherapy in liver cancer is mainly reflected in the selection of individualized treatment plan, the combination of treatment technology, the research of drug resistance mechanism, the discovery of new targets, and the improvement of immune cell function. These advances have led to more effective treatment options and a better quality of life for liver cancer patients.

### 4.4 LncRNAs and liver cancer

**Figure 3:**
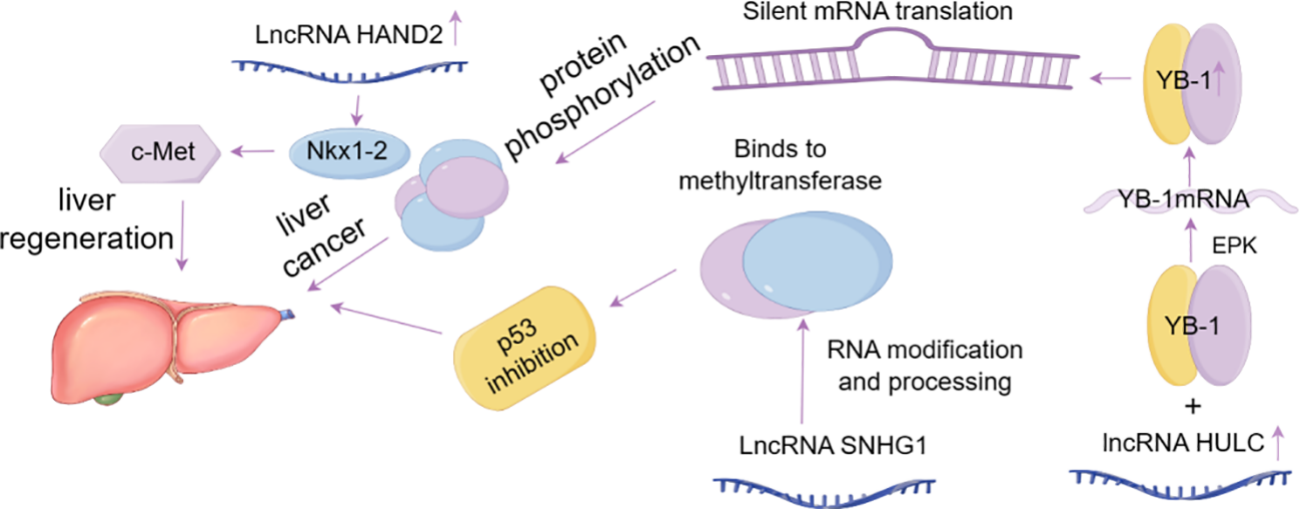
LncRNAs associated with liver cancer.

The liver is a highly regenerative and complex organ that receives all outflow circulation from the small and most of the large intestine as well as the spleen and pancreas through the portal vein. If there is temporary hepatocyte injury, the liver can regenerate rapidly within a few days to a few weeks. At the end of regeneration, the size of the hepatic lobules is significantly increased and the thickness of the hepatic cell plate is twice that of the end of regeneration (66). Various liver cancer-related lncRNAs have also been shown to have abnormal expression and can participate in cancer phenotypes by binding with DNA, RNA or proteins (**Table 1**). To date, emerging evidence points to the potential of lncRNAs to regulate ferroptosis in cancer biology. In liver cancer cells, high levels of lncRNA GABPB1 antisense RNA-1 enhance Erastin-induced ferroptosis by blocking GA-binding protein subunit beta-1 (GABPB1) translation and inhibiting peroxidoreducin-5 peroxidase. This results in the inhibition of cell antioxidant capacity and cell viability (74). For example, LncHand2 is a different RNA that is highly expressed in liver regeneration after partial hepatectomy, mainly located in the liver nucleus adjacent to the central vein of the hepatic lobule. Promotes liver regeneration by initiating transcription factor (Nkx1-2) -induced epithelial transformation factor (c-Met) signaling (71). In addition, the most studied lncRNA associated with liver cancer is HULC, which is overexpressed in liver cancer and can specifically bind YB-1 protein and accelerate its phosphorylation through extracellular signal-regulated kinase (ERK) (67), thus leading to YB-1 release from YB-1-mRNA complex and promoting the translation of silenced mRNA. It plays a carcinogenic role by regulating the phosphorylation state of its interacting proteins. Some lncRNAs, such as LncRP11-295G20.2 and LncSNHG1, are highly expressed in liver cancer cells. LncRP11-295G20.2 promotes the growth of liver cancer cells *in vitro* and *in vivo (* 68), and LncSNHG1 is mainly distributed in the nucleus of SMMC7721 cells. Usually involved in RNA processing and modification, DNMT1 is the most important methyltransferase in human body, maintaining the methyl group of newly synthesized DNA. SNHG1 promotes the development of liver cancer by inhibiting the expression of p53 through binding with DNMT1 (69) (**Figure 3**). However, some lncRNAs with dysregulated expression play a regulatory role in ferroptosis of liver cancer, and they regulate the occurrence and development of liver cancer by targeting related genes (**Table 2**).

**Table 1:** Some dysregulated LncRNAs and their roles in the progression of liver cancer.

LncRNAs	Expression	Role	Functional role	References
LncRNA DCST1-AS1	Upregulated	Promotion of liver cancer	LncRNA DCST1-AS1 mediates the occurrence and development of liver cancer through the AKT/mTOR signal transduction pathway.	(48)
LncRNA HULC	Upregulated	Promotion of liver cancer	LncRNA HULC promotes the phosphorylation of YB-1 by modulating the kinase pathway, and subsequently regulates the interaction between YB-1 and certain oncogenic mRNAs.	(67)
LncRP11-295G20.2	Upregulated	Promotion of liver cancer	LncRP11-295G20.2 binds to the N terminus of PTEN and facilitates the interaction of p62 with PTEN.	(68)
LncSNHG1	Upregulated	Promotion of liver cancer	LncSNHG1 promotes the development of liver cancer by inhibiting the expression of p53 through binding with DNMT1.	(69)
LncRNA TLNC1	Upregulated	Promotion of liver cancer	LncRNA TLNC1 interacts with TPR and induces p53 mediated by TPR, thereby inhibiting the transcription of p53 target genes and promoting the progression of liver cancer.	(70)
LncHand2	Downregulated	Inhibition of liver cancer	LncHand2 promotes liver repopulation via initiating Nkx1-2-induced c-Met signaling.	(71)
LncRNA GAS5	Downregulated	Inhibition of liver cancer	LncRNA GAS5 functioned as a tumor suppressor role in HCC through regulation of miR-21-PTEN singling pathways	(72)
LncRNA TUG1	Downregulated	Inhibition of liver cancer	LncRNA TUG1 promoted migration, invasion, and glycolysis in HCC cells via the miR-524-5p/SIX1 axis.	(73)

**Table 2:** The regulatory role of lncRNAs in modulation of ferroptosis in liver cancer.

LncRNAs	Function	Targets	Effects on tumor	References
SLC7A11-AS1	Oncogene	SLC7A11	SLC7A11-AS1 enhances HCC cells proliferation and confers resistance to erastin-induced ferroptosis by stabilizing SLC7A11 mRNA.	(75)
CASC11	Oncogene	SLC7A11	CASC11 mitigates sorafenib-induced ferroptosis by stabilizing SLC7A11.	(76)
DUXAP8	Oncogene	SLC7A11	Knocking down DUXAP8 enhances sorafenib-induced ferroptosis by promoting the de-palmitoylation of SLC7A11.	(77)
HEPFAL	Tumor suppressor	SLC7A11	HEPFAL accelerates ferroptosis by promoting the ubiquitination and degradation of SLC7A11 protein.	(78)
PVT1	Oncogene	GPX4	Overexpression of lncPVT1 and GPX4 impeded ketamine-induced ferroptosis.	(79)
HCG18	Oncogene	GPX4	Silencing HCG18 inhibits sorafenib resistance through promoting ferroptosis via inhibiting GPX4 by binding to miR-450b-5p.	(80)
URB1-AS1	Oncogene	Ferritin	HIF-1α-mediated increased URB1-AS1 attenuates sorafenib-triggered ferroptosis by inducing ferritin phase separation and decreasing the cellular free iron content.	(81)

## 5 Summary and prospect

In China, the incidence and mortality of liver cancer account for nearly 70% in Asia, and it has become a country with a high incidence of liver cancer in the world. With the progress of diagnostic technology and treatment methods, the prognosis of patients with liver cancer has been improved, but the metastasis and recurrence rates of liver cancer and the 5-year and 10-year survival rates of patients are still unsatisfactory. At present, the main treatment methods for liver cancer are traditional surgery, radiotherapy and chemotherapy, and there is still a lack of specific therapeutic means in clinical practice. However, the traditional treatment methods have limitations, and the side effects will lead to the decline of patients’ quality of life and the survival rate. Research related to early liver cancer has focused on protein-coding genes because they play a central role in the regulation of biological processes. More and more studies have shown that non-coding RNAs, especially lncRNAs, is associated with immune cell infiltration of liver cancer, and some lncRNAs signals can be integrated into the comprehensive biomarker system for immunotherapy, which has considerable potential value in improving the diagnosis and treatment level of liver cancer. LncRNAs profiles are also emerging as key regulators of genomic networks for predicting the prognosis of liver cancer. This paper mainly describes the relationship between ferroptosis, liver cancer and lncRNAs. Ferroptosis can participate in liver injury and inflammation, and lncRNAs regulates immune response, liver regeneration and REDOX signals, playing a key role in the regulation of liver microenvironment and chronic liver disease. Therefore, the mechanism of lncRNAs regulating liver cancer through ferroptosis is worth further exploration.
